# Impact of Integrated Services on HIV Testing: A Nonrandomized Trial among Kenyan Family Planning Clients

**DOI:** 10.1111/sifp.12022

**Published:** 2017-05-04

**Authors:** Kathryn Church, Charlotte E. Warren, Isolde Birdthistle, George B. Ploubidis, Keith Tomlin, Weiwei Zhou, James Kimani, Timothy Abuya, Charity Ndwiga, Sedona Sweeney, Susannah H. Mayhew, the Integra Initiative

## Abstract

The impact of integrated reproductive health and HIV services on HIV testing and counseling (HTC) uptake was assessed among 882 Kenyan family planning clients using a nonrandomized cohort design within six intervention and six “comparison” facilities. The effect of integration on HTC goals (two tests over two years) was assessed using conditional logistic regression to test four “integration” exposures: a training and reorganization intervention; receipt of reproductive health and HIV services at recruitment; a functional measure of facility integration at recruitment; and a woman's cumulative exposure to functionally integrated care across different facilities over time. While recent receipt of HTC increased rapidly at intervention facilities, achievement of HTC goals was higher at comparison facilities. Only high cumulative exposure to integrated care over two years had a significant effect on HTC goals after adjustment (aOR 2.94, 95%CI 1.73‐4.98), and programs should therefore make efforts to roll out integrated services to ensure repeated contact over time.

The integration of reproductive health (RH) and HIV services is hypothesized to have multiple service‐ and health‐related benefits. In addition to increasing cost‐effectiveness, it is expected that the co‐location of services under one roof, or within one consultation room, will minimize problematic referral processes and increase service uptake, and thus impact RH‐ and HIV‐related behaviors and outcomes (Askew and Berer 2003; Sibide and Buse 2009). Robust evidence on these potential benefits, however, is lacking. A Cochrane review on the impacts of all types of integrated primary health care found no evidence that more‐integrated services improve health‐care delivery or health status (Dudley and Garner 2011). Reviews on the integration of RH and HIV services, specifically, conclude that research evidence on outcomes is lacking, with few studies adequately defining and measuring integrated services, or comparing integrated with stand‐alone health services (Kennedy et al. 2010; Lindegren et al. 2012; Wilcher et al. 2013).

One potential benefit of integrated care is increased utilization of the individual component health services. Increasing the uptake of HIV testing and counseling (HTC) is a critical public health goal, since the proportion of adults who know their HIV status rarely exceeds 50 percent in most high‐ and medium‐HIV prevalence settings (UNAIDS 2013 and 2014b). Annual testing rates are even lower, with national surveys reporting only around one‐fifth of women and men receiving a test in the past year (Staveteig et al. 2013). Knowledge of HIV status is an essential prerequisite to accessing antiretroviral therapy (ART) for people living with HIV (PLHIV), and the need to scale up testing has been asserted in new “90‐90‐90” global targets, aiming to have 90 percent of PLHIV knowing their status, 90 percent on sustained ART, and 90 percent with viral suppression by the year 2020 (UNAIDS 2014a). Knowledge of status also contributes to HIV prevention, not only through access to treatment and associated viral suppression, but through reductions in the risk of perinatal and onward sexual transmission (Denison et al. 2008; Kennedy et al. 2013).

Repeated testing every 6 to 12 months has been recommended by the World Health Organization since 2007 for those at higher risk of HIV exposure. In Kenya, where HIV prevalence is estimated at 6.1 percent (UNAIDS 2013) and risk of exposure is high, repeat annual testing for those who test negative has been recommended since 2010 (NASCOP 2010). However, a national household survey indicated that only 29 percent of women and 23 percent of men have tested in the past 12 months (Staveteig et al. 2013). Integration between RH and HIV services has been rolled out nationally as a strategy to promote HIV testing by the Kenyan Ministry of Health (MOPHS 2009 and 2012).

Multiple strategies have been designed and evaluated to promote uptake of HTC within generalized epidemics. Provider‐initiated testing and counseling for HIV is one intervention that has shown proven impact on HIV testing uptake when services were integrated within antenatal care, primary care, STI, and TB services (Pope et al. 2008; Leon et al. 2010; Kennedy et al. 2013). A systematic review on the implementation of provider‐initiated testing and counseling in sub‐Saharan Africa, however, found challenges with the approach, with levels of test offering and acceptance varying markedly by study setting (Roura et al. 2013). For maternal and child health (MCH) programs, promoting HTC within antenatal care has remained a focus as it is an essential strategy to prevent mother‐to‐child transmission (Baggaley et al. 2012), and it is being increasingly promoted through the roll‐out of the Option B+ regimen (Herlihy et al. 2015). Documentation of the integration and promotion of HTC within family planning (FP) services is more limited. FP clients are an important target group for testing since they are sexually active and usually not current condom users. Evidence on the effectiveness of integrating HTC into FP services is limited. One cross‐sectional analysis of FP clinic records in Ethiopia compared integration at provider, room, and facility levels (i.e., assessing whether HTC uptake differed when offered by the same provider, in the same room, or in the same building as the FP service) (Bradley et al. 2008). Higher HIV testing uptake was found in facilities with room‐ and provider‐level integration. In Kenya's Central Province, an uncontrolled pre/post‐test comparing an “integrated” FP‐HTC model (on‐site testing) with a “referral” model found increases in discussion of HIV during consultations and increases in HIV testing acceptance following a training and counseling support intervention, with testing acceptance higher in the “on‐site” testing group (Liambila et al. 2009). However, no attempt was made to control for any selection bias in the two study populations and the evaluation was conducted over a short period (ten months).

In their Cochrane review on integrated care, Dudley and Garner (2011) underline the complexity in the definition and measurement of integrated care, and the need for clear and transparent documentation of any integrated intervention being evaluated. In general, most interventions involve some degree of care reorganization, but others have merely provided training and/or the provision of equipment (Kennedy et al. 2010; Dudley and Garner 2011). Most fail to assess whether integrated care (linked provision by one provider, or at one visit) is actually being provided to the client, and outcomes may be associated with interventions that were not fully implemented.

In this article, we assess the impact of integrating HIV and FP services on the HTC uptake of FP clients in Central Province, Kenya and specifically test the effect of four different exposure definitions of integrated care: an intervention involving training and reorganization; receipt of both RH and HIV services at recruitment; a functional measure of facility integration at recruitment; and a woman's cumulative exposure to functionally integrated care across different facilities over time. The research was conducted as part of the Integra Initiative, a large‐scale evaluation of RH‐HIV service integration in Kenya and Swaziland. The Integra Initiative is a registered nonrandomized trial.^1^ Integra aims to evaluate the effect of service integration within FP and postnatal‐care settings and is comprised of multiple quantitative and qualitative components, including household surveys, cohort studies, facility surveys, and qualitative process evaluation (Warren et al. 2012).

## METHODS

### Study Setting and Design

Integra was implemented in public health facilities in Central and Eastern Provinces in Kenya, and in three regions in Swaziland. Our article focuses on findings from Central Province in Kenya, where an intervention was introduced into six facilities (health centers and hospitals) to strengthen the provision of integrated FP‐HIV services. Compared to the national average, at the time of the research the region had a higher modern contraceptive prevalence (46 percent versus 67 percent) (NBS Kenya 2007) and lower HIV prevalence (5.6 percent versus 3.8 percent) (NASCOP 2007).

Integra originally sought a controlled pre/post‐test (quasi‐experimental) design to measure the effect of integrated health care in intervention sites. Due to challenges in ensuring program implementation in intervention sites, and the existence of non‐Integra integration activities in “control” sites, the latter are referred to as “comparison sites,” and in this article we treat the whole sample as a cohort to assess the effect of individuals’ exposure to integrated care on HIV testing outcomes. The cohort was female FP clients (aged 15–49 years) attending the six intervention and six comparison facilities. The facilities included six hospitals and six health centers. Characteristics of study facilities are described in Table 1.

**Table 1:** Characteristics of study facilities (pre‐intervention), by design group

Design group	Facility code^a^	Type of facility	Location	Catchment population	Total FP clients (2009)	Number of nurse/ midwives in MCH^b^	Integration structure in 2009 (pre‐intervention)^d^	Pair match facility code
Intervention	23	District hospital	Urban (city)	560,230	7,402	14	HTC in FP room	4
	3	Provincial hospital	Urban (town)	46,707	5,804	7	HTC in FP room	6
	10	Health center	Peri‐urban (edge of town)	69,363	5,723	6	HTC in FP room	9
	21	Sub‐district hospital	Urban (town)	46,707	2,871	1	HTC in FP room	25
	14	Health center	Rural	7,680	1,925	3	HTC within MCH unit, sometimes in same room	13
	2	Health center	Rural	23,000	2,245	0^c^	HTC in separate room	5
**Comparison**	4	District hospital	Urban (town)	53,541	5,257	8	HTC in FP room	23
	6	District hospital	Urban (town)	308,000	4,529	3	HTC in FP room	3
	9	District hospital	Rural	21,525	2,245	3	HTC within PMTCT, not in MCH unit	10
	25	Health center	Rural	23,516	1,422	3	HTC in FP room (part of PITC) initiative	21
	13	Health center	Rural	29,880	3,541	6	HTC within MCH unit	14
	5	Health center	Rural	12,294	2,372	6	HTC within MCH unit	2

a Code referred to in Mayhew et al. 2016.

b Registered or enrolled.

c Only one clinical officer reported.

d Intervention facilities had previously received integration support.

FP = Family Planning. HTC = HIV testing and counseling. MCH = Maternal and Child Health. PITC = Provider‐initiated testing and counseling for HIV. PMTCT = Prevention of mother‐to‐child‐transmission of HIV.

SOURCE: Integra Periodic Activity Review 2009 (structured tool capturing data on service characteristics and staffing).

Intervention sites were selected based on good performance in a previous integration study (Liambila et al. 2009), were located in districts that were early implementers of national FP‐HIV integration policy, and had high client load (≥100/month). Comparison sites were located in districts that had not yet implemented national FP‐HIV integration policy, and were selected using a pair‐wise matching design, with matching based on client load, number of providers qualified and currently delivering FP services, and range of services available. Facilities were selected in different districts of the same province to minimize contamination.

The study intervention is described in detail elsewhere (Warren et al. 2012), but in short it was designed to add the following services into standard FP service delivery: discussion of fertility desires, condom promotion/provision, STI/HIV risk assessment, HIV status check, HTC provision, cervical cancer screening, pre‐HIV treatment services and/or referral to HIV treatment unit for HIV‐positive clients. The provision of these services was supported by training on and provision of an integrated client counseling toolkit, the “Balanced Counseling Strategy Plus” (BCS+) (Population Council 2016). In addition, intervention facilities were supported by nurse/midwife “mentors” who received training on SRH/HIV technical skills to provide mentorship and supportive supervision on integrated care to others on‐site (Ndwiga et al. 2014). The layout of some clinics was also reorganized to support integrated care provision, and essential equipment and supplies were provided to deliver integrated services.

### Data Collection

FP clients were recruited between the end of 2009 and early 2010, and interviewed at four time points over two years: baseline (r0) (immediately after intervention implementation), round 1 (r1) (r0+6 months); round 2 (r2) (r0+18 months); and round 3 (r3) (r0+24 months). The recruitment interview took place at the health facility using a structured questionnaire on a personal digital assistant (PDA), and subsequent interviews were conducted either at the respondent's home or at an arranged meeting at the health facility, also using PDAs. The questionnaire was in Kiswahili and collected data on socio‐demographic characteristics, family planning practices, HIV‐related behaviors and practices, service‐use history, and perceptions of service quality. Respondents gave their informed consent before each interview.

At recruitment, clients were sampled consecutively as they exited consultations. Sample size calculations were based on having 80 percent power to detect an absolute between‐group increase from 5 percent to 10 percent in another study outcome (consistent condom use) among those using other contraceptive methods. Based on condom use estimates in a previous study (Liambila et al. 2009; Mwangi and Warren 2008) and with a significance level of 5 percent, it was estimated that 1,952 participants would be needed, assuming a 30 percent loss to follow‐up.

### Study Population

Of the original recruitment sample (N=1,958), the following women were excluded sequentially from the analysis: 245 known to be HIV‐positive at recruitment, tested either before or during recruitment consultation (139 in intervention [14 percent] and 106 in comparison [11 percent]); 745 without a complete cohort data history (r0 through r3) (345 in intervention [41 percent] and 400 in comparison [46 percent]); and 86 missing complete data on all potentially confounding variables (64 in intervention [13 percent] and 22 in comparison [5 percent]), resulting in a sample size of 882 for a complete case analysis.

### Measuring the Uptake of HIV Testing

At every round, respondents were asked whether they had received an HIV test—during consultation at recruitment, or since their last interview in subsequent rounds—and the date of the test. Participants who remained HIV‐negative (as self‐reported in cohort interviews) and received at least two HIV tests over the two‐year cohort period were considered to have fulfilled the outcome, “HTC goals achieved,” since annual testing is the national recommendation in Kenya (NASCOP 2010). Those who reported a positive HIV test during the study were categorized as “HTC goals achieved” if they reported at least one HIV test during the study.

### Measures of RH‐HIV Integration

We investigated the impact of service integration on HTC uptake using four different measures of integrated care. The different approaches, summarized in Table 2, reflect different a priori questions and mechanisms—at both the facility and individual level—by which integration may influence client outcomes, and the fact that there are no standard definitions of integration in research or health practice.

**Table 2:** Different measures used to define integrated care

Research question	Integration exposure measure	Definition	Notes
1) Does the Integra Intervention have an effect on HIV testing uptake among FP clients, compared with FP clients in facilities that did not receive the Integra intervention?	Design group	Attended intervention or comparison facility at the time of recruitment visit	6 intervention, 6 comparison facilities
2) Does the receipt of integrated RH‐HIV services during an FP visit increase annual HIV testing over the subsequent two years (regardless of study arm)?	Individual receipt of integrated services at baseline	Woman received a combination of at least one RH service (FP, MCH) and one HIV/STI service (HIV testing, HIV counseling, STI service) during her consultation at baseline	Binary measure (yes/no). HIV testing uptake measured at Rounds 1–3 only (baseline excluded)
3) Does the level of integration at the facility lead to an increase in annual HIV testing among FP clients (regardless of study arm)?	Baseline facility integration index score	Score derived from Integra Functional Integration Index (IFII) to measure the extent of integration at the facility level	Low (≤1.99), medium (2.00 to 2.74), or high (≥2.75) index integration score
4) Does the cumulative score for the level of integration in all facilities visited by a woman over two years influence her uptake of annual HIV testing?	Cumulative integration index score	Cumulative index exposure (additive score) to capture subsequent visits at study clinics	Grouped by tertiles of cumulative score into low, medium, and high.

The first measure, “Design group,” categorized women by the study arm (per protocol), based on whether the facility from where they were recruited was designated as an intervention or comparison site. This maintains the original quasi‐experimental approach.

Subsequent exposures use a cohort study design. The second measure captured each individual woman's actual receipt of integrated services during the recruitment consultation, irrespective of the facility's designation as intervention or comparison site in the study. Services used were self‐reported by women in the exit interview. “Integrated services” were defined as a visit in which a woman received at least one RH service (FP counseling, FP method provision/check‐up, postnatal care for mother, postnatal care for baby, child health, or cervical cancer screening) AND at least one HIV/STI service (STI counseling or treatment, HIV counseling, HIV test, HIV treatment and care, psycho‐social support for HIV, treatment of opportunistic infections, TB service). Since HIV testing forms a part of this definition, the outcome measure for this exposure was restricted to HIV tests received in rounds 1–3 only (excluding r0).

The third measure recorded the degree of integrated care being delivered at the facility at recruitment, as measured by the Integra Functional Integration Index. The Integra Index is a multidimensional score of facility‐level integration derived from data collected through client flow analyses and calculated using latent variable modeling (Mayhew et al. 2016). The Index measures integration as a continuum, so that differences in the extent and nature of integration across facilities can be understood. It is derived from four indicators capturing the extent to which a facility's clients receive both RH and HIV services during their visits. Index scores at recruitment ranged from 0.87 to 3.42 across the 12 facilities; they were categorized into low (≤1.99), medium (2.00–2.74), or high (≥2.75) index integration scores, based on the distribution of the data.

Since women may return for FP consultations differentially, or switch facilities (i.e., visit facilities other than the recruitment facility) during the two‐year follow‐up period of the study, and the extent of integration at a facility can vary over time, the fourth measure recorded a cumulative integration score that took into account each woman's individual use of integrated clinics throughout the study (as self‐reported by women in each cohort interview). To calculate the cumulative score, Index scores were summed for every FP visit reported over the two‐year cohort period, although scores for visits to non‐Integra study facilities were not captured (14 percent and 16 percent of FP visits at r2 and r3, respectively). The cumulative exposure score was grouped into three categories—low, medium, and high—based on tertiles of the data (since scores were more evenly distributed across the far wider range of individual scores).

### Statistical Analyses

Data were imported into STATA 13.0 for cleaning, checking, and analysis. We used z tests for differences in proportions in HIV testing between cohort rounds, and chi squared tests for crude associations between HIV testing and each of the four measures of integration, as well as between potentially confounding variables and outcome.

**Figure 1:**
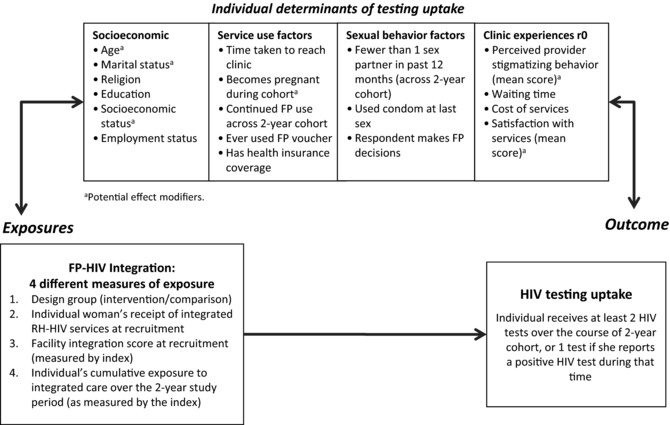
Conceptual framework identifying potential mediators of the relationships between integration and HIV testing uptake

Potential confounders were identified through a review of the literature of factors influencing HIV testing uptake (Kalichman and Simbayi 2003; Zeelie, Bornman, and Botes 2003; Fylkesnes and Siziya 2004; Warwick 2006; Nakanjako et al. 2007; Musheke et al. 2013), and are displayed in the conceptual framework in Figure 1. Most of these factors were measured at r0, though selected indicators were recorded at every round (“Becomes pregnant,” “Continued use of FP,” “More than one sex partner in past 12 months [at any time]”). Socioeconomic status groupings were based on a principal components analysis of household assets. The “provider stigma score” was based on a mean score derived from Likert scales (1–5) on client reports on the following clinic characteristics: privacy, confidentiality of consultation, trust in records being kept confidential, and people living with HIV (PLHIV) treated same as others. “Satisfaction with services” was based on a mean score derived from Likert scales (1–5) on: overall service rating, costs, waiting time, availability of drugs and supplies, possibility of receiving other services at the same time, opening times, provider friendliness, doctor/nurse availability, providers listened, client could ask questions.

Multivariable analyses were conducted to test the association between integration exposure and achievement of HTC goals, to control for potential confounding. We used conditional logistic regression models to account for clustering at the facility level, including all potential confounders in the model (i.e., theory‐driven selection of variables). Potential effect modification of the relationship between integration and HIV testing by certain variables (identified conceptually, see Figure 1) was tested using the Mantel‐Haenszel method, but no effect modification was found (data not shown).

### Sensitivity Analyses

To examine the effect of facility pair‐matching, we also constructed a conditional regression model using the STATA svy commands to account for clustering within matched pairs, in addition to the conditional dependency on the facility cluster. Analyses that allowed for such clustering (by facility pairs) gave almost identical results to those that assumed independence, and the latter are reported.

Sensitivity analyses were also conducted for missing data on complete cases. We conducted χ2 statistical tests to assess how those with complete cohort data differed from those with an incomplete cohort history across baseline exposure and potential confounding factors. Those with complete data differed from those who were excluded from the analysis in age (p<0.001, with those in the youngest age group less likely to have complete data), and whether they paid fees for services (p=0.017, those paying fees were less likely to have complete data). There was no evidence of a difference in other baseline characteristics or between clinics. The implications of these differences are addressed in the discussion.

## RESULTS

Table 3 displays the socio‐demographic and service‐related characteristics of the study population, in aggregate and by design group. Women in intervention clinics were younger (17 percent were over 35, versus 28 percent at comparison sites), had similar marital patterns (97 percent of both groups were married), had different religious beliefs (with fewer Pentecostals (32 percent versus 40 percent), were more highly educated (10 percent had received some tertiary education versus 3 percent in comparison sites), had higher self‐employment (46 percent versus 37 percent) and lower manual employment (6 percent versus 16 percent), and lived further from their clinic (45 percent lived more than 30 minutes away versus none in comparison sites). In terms of SRH behaviors, the groups had similar probabilities of becoming pregnant or continuing FP over the cohort (16 percent versus 12 percent, and 80 percent versus 83 percent, respectively); intervention participants were less likely to use a voucher for FP (3 percent versus 6 percent), but had similar health insurance (22 percent overall). Multiple sexual partnerships were commonly low across the groups (2.4 percent overall), as was condom use at last sex (3.4 percent). Women in intervention clinics were more likely to make decisions concerning FP than those in comparison clinics (57 percent versus 47 percent). They were more likely to be dissatisfied with services (10 percent versus 5 percent) and to have waited longer (57 percent had to wait more than 30 minutes, versus 0.2 percent in comparison sites), though they were less likely to have paid fees for services (83 percent versus 93 percent).

**Table 3:** Socio‐demographic and health service characteristics of study sample, by design group

	Comparison		Intervention		Total		
Characteristic	N	(%)	N	(%)	N	(%)	P value (χ2)
**Age group**							
Under 25	114	(25.7)	117	(26.7)	231	(26.2)	<0.001
25–29	109	(24.6)	142	(32.3)	251	(28.5)	
30–34	95	(21.4)	107	(24.4)	202	(22.9)	
35–39	85	(19.2)	52	(11.8)	137	(15.5)	
40 and over	40	(9.0)	21	(4.8)	61	(6.9)	
**Marital status**							
Single or has boyfriend/partner	8	(1.8)	9	(2.1)	17	(1.9)	0.904
Married	431	(97.3)	427	(97.3)	858	(97.3)	
Divorced/separated/widowed	4	(0.9)	3	(0.7)	7	(0.8)	
**Religion**							
Protestant	138	(31.2)	155	(35.3)	293	(33.2)	0.050
Roman Catholic	104	(23.5)	125	(28.5)	229	(26.0)	
Pentecostal	179	(40.4)	141	(32.1)	320	(36.3)	
Other/None	22	(5.0)	18	(4.1)	40	(4.5)	
**Education (highest level)**							
None/Primary	286	(64.6)	236	(53.8)	522	(59.2)	<0.001
Secondary	144	(32.5)	161	(36.7)	305	(34.6)	
Tertiary	13	(2.9)	42	(9.6)	55	(6.2)	
**Socio‐economics status score (quintiles)**							
1st (poorest)	117	(26.4)	66	(15.0)	183	(20.7)	<0.001
2nd	99	(22.3)	78	(17.8)	177	(20.1)	
3rd	93	(21.0)	82	(18.7)	175	(19.8)	
4th	79	(17.8)	93	(21.2)	172	(19.5)	
5th (wealthiest)	55	(12.4)	120	(27.3)	175	(19.8)	
**Employment status**							
Student/Unemployed	160	(36.1)	152	(34.6)	312	(35.4)	<0.001
Casual worker/Informal sector	32	(7.2)	40	(9.1)	72	(8.2)	
Employed (manual)	70	(15.8)	24	(5.5)	94	(10.7)	
Self‐employed	162	(36.6)	201	(45.8)	363	(41.2)	
Employed (professional/technical)	19	(4.3)	22	(5.0)	41	(4.6)	
**Time to reach clinic (minutes)**							
0–30	443	(100.0)	241	(54.9)	684	(77.6)	<0.001
31–60	0	(0.0)	123	(28.0)	123	(13.9)	
>60	0	(0.0)	75	(17.1)	75	(8.5)	
**Became pregnant during cohort**	53	(12.0)	68	(15.5)	121	(13.7)	0.128
**Continued FP through cohort**	369	(83.3)	350	(79.7)	719	(81.5)	0.172
**Used FP voucher**	28	(6.3)	13	(3.0)	41	(4.6)	0.018
**Health insurance**	89	(20.1)	105	(23.9)	194	(22.0)	0.170
**Multiple sexual partners (any cohort round)**	12	(2.7)	9	(2.1)	21	(2.4)	0.521
**Condom use at last sex**	15	(3.4)	15	(3.4)	30	(3.4)	0.980
**Who makes FP decisions?**							
Woman decides	207	(46.7)	252	(57.4)	459	(52.0)	0.003
Partner or provider decides	53	(12.0)	32	(7.3)	85	(9.6)	
Both agree/other	183	(41.3)	155	(35.3)	338	(38.3)	
**Provider stigmatizing behavior perception (r0)**							
Low	88	(19.9)	182	(41.5)	270	(30.6)	<0.001
Medium	335	(75.6)	233	(53.1)	568	(64.4)	
High	20	(4.5)	24	(5.5)	44	(5.0)	
**Satisfaction with services (r0)**							
High	118	(26.6)	131	(29.8)	249	(28.2)	0.004
Medium	304	(68.6)	265	(60.4)	569	(64.5)	
Low	21	(4.7)	43	(9.8)	64	(7.3)	
**Paid fees for services (r0)**	411	(92.8)	362	(82.5)	773	(87.6)	<0.001
**Waiting time**							
≤30 mins	442	(99.8)	190	(43.3)	632	(71.7)	<0.001
>30 mins	1	(0.2)	249	(56.7)	250	(28.3)	
**Total**	**443**	**(100.0)**	**439**	**(100.0)**	**882**	**(100.0)**	

**Figure 2:**
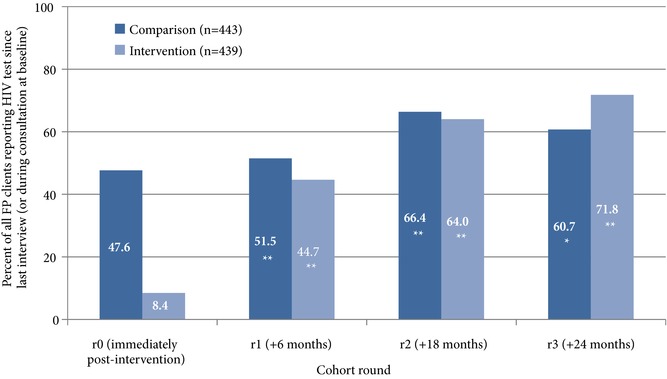
Proportion who reported receiving an HIV test since last interview, by round and design group Difference from previous round: **p<0.01; *p<0.05.

Overall, 69.3 percent of women achieved HIV testing goals over the two‐year cohort. Thirty percent received two HIV tests, 28 percent received three, and 10 percent received four (data not shown). Reports of HIV testing increased markedly over the course of the cohort, from 28 percent at baseline (during consultation) to 48 percent at r1, 65 percent at r2, and 66 percent at r3 (all reported as test since last interview) (data not shown). In the comparison facilities, an average of 48 percent of participants received an HIV test at baseline (see Figure 2). This increased slightly to 52 percent at r1 and jumped to 66 percent at r2 before a slight reduction to 61 percent in the final round. In contrast, participants in intervention facilities reported far lower levels of HIV testing at recruitment (8 percent). By r1, reports jumped significantly to 45 percent and continued to increase in r2 (to 64 percent) and in r3, when 72 percent of women recruited in intervention facilities reported receiving an HIV test since their last interview.

**Figure 3:**
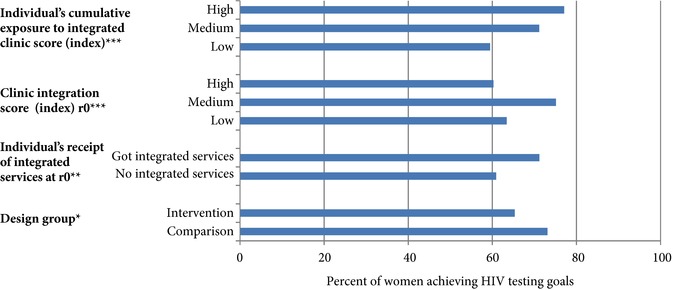
Percent of women achieving HIV testing goals over the two‐year cohort, by different exposure groups (n=882) Chi‐squared test: *p<0.05; **p<0.01; ***p≤0.001.

Figure 3 displays HIV testing outcomes by the four different exposure measures. Over the course of the study, more women in the comparison group (73 percent) met the HIV testing goal compared to the intervention group (65 percent). Women who received integrated services at baseline—regardless of design group—were more likely to receive the two‐test minimum (after r0) (71 percent) compared to those who did not (61 percent). There was no clear association with baseline integration index score, with those visiting a facility with a “medium” integration score most likely to receive the test outcome (75 percent) versus the high (60 percent) and the low (63 percent) groups. There was a clearer association with the cumulative integration index score, with those women having the highest cumulative exposure to integrated services most likely to have received the testing requirement (77 percent) versus the medium‐score group (71 percent) and the low‐score group (60 percent).

For the two exposure measures with a crude positive impact of integration (individual's receipt of integration at baseline and cumulative clinic integration score), we further tested these associations through multivariable analyses. Association between individual receipt of integration and achievement of HTC goals became nonsignificant after adjustment (aOR 1.38, 95%CI 0.88‐2.18) (data not shown). For the cumulative clinic integration score exposure, both crude and adjusted associations between integration and HTC goals, as well as with other socio‐demographic and service‐related covariates, are presented in Table 4. After adjustment, strong evidence remained of the association between cumulative exposure to integrated clinics and HTC goal achievement: those with medium exposure to clinic integration had nearly double the odds of achieving HTC goals than those in the low‐exposure group (aOR 1.92, 95%CI 1.24‐2.97), and those in the highest exposure group had nearly three times the odds of HTC goal achievement (aOR 2.94, 95%CI 1.73‐4.98). Few other covariates were associated with testing uptake (see grey shading). There was weak evidence that women becoming pregnant subsequent to r0 had higher odds of testing uptake (aOR 1.97, 95%CI 0.95‐2.68), and those who had health insurance at r0 were also more likely to report testing uptake (aOR 1.59, 95%CI 1.05‐2.50).

**Table 4:** Multivariable results of association between cumulative integration index score and HIV testing outcome (n=882)

		HIV testing					
Variable/category	N	N	%	cOR	95%Cl	aOR^a^	95%CI
**Cumulative exposure to integration score**							
Low	294	175	(59.5)	1.00		1.00	
Medium	295	210	(71.2)	1.68	(1.19–2.37)	1.92	(1.24–2.97)
High	293	226	(77.1)	2.29	(1.60–3.28)	2.94	(1.73–4.98)
**Age group**							
Under 25	231	169	(73.2)	1.00		1.00	
25–29	251	164	(65.3)	0.69	(0.47–1.02)	0.73	(0.47–1.12)
30–34	202	138	(68.3)	0.79	(0.52–1.20)	0.88	(0.56–1.41)
35–39	137	96	(70.1)	0.86	(0.54–1.37)	0.70	(0.41–1.21)
40 and over	61	44	(72.1)	0.95	(0.51–1.78)	0.65	(0.30–1.41)
**Marital status**							
Single or has boyfriend/partner	17	15	(88.2)	3.39	(0.77–14.92)	3.30	(0.65–16.72)
Married	858	591	(68.9)	1.00		1.00	
Divorced/separated/widowed	7	5	(71.4)	1.13	(0.22–5.86)	1.81	(0.29–11.16)
**Religion**							
Protestant	293	203	(69.3)	1.00		1.00	
Roman Catholic	229	163	(71.2)	1.09	(0.75–1.60)	1.21	(0.79–1.85)
Pentecostal	320	216	(67.5)	0.92	(0.65–1.30)	0.84	(0.57–1.25)
Other/None	40	29	(72.5)	1.17	(0.56–2.44)	0.99	(0.42–2.32)
**Education**							
None/Primary	522	374	(71.6)	1.00		1.00	
Secondary	305	203	(66.6)	0.79	(0.58–1.07)	0.80	(0.56–1.16)
Tertiary	55	34	(61.8)	0.64	(0.36–1.14)	0.83	(0.40–1.74)
**Socio‐economic status quantile**							
1st (poorest)	183	135	(73.8)	1.00		1.00	
2nd	177	124	(70.1)	0.83	(0.52–1.32)	1.33	(0.78–2.27)
3rd	175	129	(73.7)	1.00	(0.62–1.60)	1.68	(0.95–2.99)
4th	172	110	(64.0)	0.63	(0.40–0.99)	1.37	(0.76–2.45)
5th (wealthiest)	175	113	(64.6)	0.65	(0.41–1.02)	1.55	(0.81–2.96)
**Employment status**							
Student/Unemployed	312	213	(68.3)	1.00		1.00	
Casual worker/Informal sector	72	45	(62.5)	0.77	(0.45–1.32)	0.74	(0.41–1.36)
Employed (manual)	94	73	(77.7)	1.62	(0.94–2.77)	0.82	(0.42–1.62)
Self‐employed	363	256	(70.5)	1.11	(0.80–1.54)	0.84	(0.56–1.25)
Employed (professional/technical)	41	24	(58.5)	0.66	(0.34–1.28)	0.67	(0.30–1.50)
**Distance from clinic (minutes)**							
0–30	684	473	(69.2)	1.00		1.00	
31–60	123	77	(62.6)	0.75	(0.50–1.11)	0.96	(0.58–1.59)
>60	75	61	(81.3)	1.94	(1.06–3.55)	1.33	(0.61–2.88)
**Became pregnant over cohort**							
No	761	522	(68.6)	1.00		1.00	
Yes	121	89	(73.6)	1.27	(0.83–1.96)	1.59	(0.95–2.68)
**Continued use of FP over cohort**							
No	163	102	(62.6)	0.69	(0.48–0.98)	0.78	(0.50–1.24)
Yes	719	509	(70.8)	1.00		1.00	
**Used FP voucher**							
No	841	578	(68.7)	1.00		1.00	
Yes	41	33	(80.5)	1.88	(0.86–4.12)	1.30	(0.53–3.18)
**Has health insurance**							
No	688	475	(69.0)	1.00		1.00	
Yes	194	136	(70.1)	1.05	(0.74–1.49)	1.62	(1.05–2.50)
**Multiple sex partners**							
No	861	594	(69.0)	1.00		1.00	
Yes	21	17	(81.0)	1.91	(0.64–5.73)	1.88	(0.53–6.65)
**Condom use at last sex**							
No	852	590	(69.2)	1.00		1.00	
Yes	30	21	(70.0)	1.04	(0.47–2.29)	1.58	(0.65–3.88)
**Who makes FP decisions**							
Woman decides	459	313	(68.2)	1.00		1.00	
Partner/provider decides	85	63	(74.1)	1.34	(0.79–2.25)	1.27	(0.70–2.31)
Both agree/other	338	235	(69.5)	1.06	(0.79–1.44)	1.11	(0.78–1.57)
**Provider stigmatizing behavior r0**							
Low	270	192	(71.1)	1.00		1.00	
Medium	568	385	(67.8)	0.85	(0.62–1.17)	0.81	(0.54–1.22)
High	44	34	(77.3)	1.38	(0.65–2.93)	1.22	(0.48–3.10)
**Satisfaction score r0**							
High	249	175	(70.3)	0.66	(0.34–1.27)	0.94	(0.42–2.09)
Medium	569	386	(67.8)	0.59	(0.32–1.10)	0.87	(0.42–1.83)
Low	64	50	(78.1)	1.00		1.00	
**Paid fees r0**							
No	109	82	(75.2)	1.40	(0.88–2.22)	0.92	(0.48–1.76)
Yes	773	529	(68.4)	1.00		1.00	
**Waiting time r0**							
≤30 mins	632	447	(70.7)	1.00		1.00	
>30 mins	250	164	(65.6)	0.79	(0.58–1.08)	1.31	(0.82–2.08)

a Adjusted for all other variables in table.

## DISCUSSION

This analysis demonstrates the complexity of assessing the effect of health service re‐organization on health and behavioral outcomes. The results show that determining whether “service integration” impacts uptake of HIV testing depends on *how* “integration” is measured. Findings also point to the need to articulate a precise definition of the type of integrated service‐delivery that is occurring at any given clinic if meaningful interpretation is to be achieved.

An integration intervention had a positive effect on initially increasing HIV testing uptake from very low levels immediately post‐intervention, as the proportion of FP clients at these facilities who received an HIV test increased dramatically over the course of the study, particularly in the first six months after the intervention. In contrast, the “comparison” sites provided much higher levels of HIV testing at r0, and levels rose moderately over time. The dramatic increase in HIV testing in intervention facilities replicates positive results reported in a previous uncontrolled study of a similar intervention in Central Province, Kenya (Liambila et al. 2009). The greater increase in intervention clinics relative to comparison sites suggests that the BCS+ toolkit was effective in encouraging providers to promote HIV testing, in particular where there was a low baseline and potential latent demand for testing (Population Council 2016). The BCS+ toolkit is an evidence‐based, interactive, client‐friendly approach that aims to improve contraceptive counseling by addressing a variety of topics relevant to FP including prevention, detection/testing, and treatment of HIV and STIs; postpartum maternal and newborn care; and cervical cancer screening. Other provider job aids have been found to be effective in supporting integration activities, including screening tools and flip‐charts (Kim et al. 2005; Foreit 2006; Baumgartner et al. 2014), and programs should continue to support their use to broaden the scope of health consultations. The costs of production of and training on job aids can seem prohibitive to programs, but the existence of proven global or national tools should make their adaptation, implementation and/or dissemination easier. In Kenya, Integra was able to review and update existing MOH job aids. Another report from Integra has also pointed to the important role mentors played during the intervention, helping to improve provider knowledge, skills, self‐confidence, and teamwork (Ndwiga et al. 2014).

We found no difference, however, between attendance at intervention clinics and achievement of total testing goals over the two‐year study period, likely due to markedly lower r0 levels of testing in intervention sites. One explanation for lower levels of HIV testing at intervention sites at r0 may have been previous receipt of HIV testing, potentially resulting from the previous integration support (Liambila et al. 2009). This is compounded by the fact that prior to 2010 retesting guidance was unclear, and annual testing was not made explicit. Since our questionnaire only recorded past testing history among those who received an r0 test, it is not possible to contrast or control for baseline differences; but among those whose history was recorded, testing levels were indeed far higher in intervention than control sites (89.8 percent versus 50.8 percent). In addition, adhoc program changes may have blurred the categorization by design groups: in intervention sites there were challenges with test‐kit stock‐outs and in‐staff rotation, limiting intervention activities, and in comparison sites initiatives from the Ministry of Health and partner agencies were encouraging HTC for the three months around recruitment. Another Integra analysis, developing the Integra Indexes, demonstrated that integration scores (i.e., the provision of multiple services by one provider, within one consultation or within one visit) did not correlate with design group and thus the relatively large observed increase in testing uptake at intervention sites after r0 should be interpreted with caution (Mayhew et al. 2016). This and other Integra analysis indicated that health‐facility structures need to be prepared with equipment and training before integration activities can occur, but these structural inputs are not in themselves sufficient to achieve integrated service delivery—which will depend on staff action, motivation, and support (Mayhew et al. 2016; Mayhew et al., forthcoming).

To answer the question of whether “integrated services” have an effect on testing goals, it has been informative to also investigate the impact of integration measured in other ways. We found a crude effect on HTC uptake of an individual's receipt of integrated RH‐HIV services at baseline, but not after adjustment. An individual's cumulative exposure to integrated clinics over time, as measured by the multidimensional index of integration was, however, still significantly associated with testing uptake after adjustment for confounding. This implies that women who return frequently for FP services to more integrated clinics are more likely to receive their recommended HIV tests than women who return frequently to less integrated clinics. Family planning services often require follow‐up visits, but efforts may be required to encourage women to return who have either discontinued or opted for long‐term reversible or permanent contraceptive methods. Follow‐up visits would have the beneficial impact of encouraging engagement with both the FP and HIV service components.

It was also surprising that so few other socio‐demographic or behavioral factors were associated with testing uptake. Women who became pregnant were more likely to get tested, reflecting the provision of HTC in antenatal care. Interestingly, having health insurance was associated with testing goals, which is surprising given the supposedly free provision of HTC and ART in Kenya. Insurance may be promoting the use of other services, however, which then provide the opportunity for testing promotion. Other factors that might have been expected to influence HTC, such as perceived provider stigma, distance living from a testing site, socio‐economic status, and age (Obermeyer and Osborn 2007; Musheke et al. 2013), all had no influence. The fact that this analysis investigated the receipt of at least two tests over a two‐year period may explain this difference, since existing studies have focused on uptake of a first HIV test. Repeat testing is therefore seemingly more heavily influenced by clinic‐level factors, and this analysis shows that repeated integration exposure is one of them. This therefore provides a strong rationale for national health programs to respond by scaling up the integration of HIV testing into FP services.

In addition to problems associated with categorization by design group noted above, this analysis has other important limitations. First, the quasi‐experimental design implies risk of selection bias. Unmeasured confounding from other factors affecting testing uptake is plausible, and in particular the failure to control to past testing history, as noted above, may have influenced findings. Other factors that we could not control for, but which have been shown to influence testing uptake include perceived availability of ART at the clinic, perceived risk of HIV infection, physical health symptoms, and death of a sexual partner and/or child (Musheke et al. 2013). Nevertheless, most of these other factors would not be expected to vary by clinic. Perceived availability of ART is implicitly linked to clinic integration score, and therefore could not be included independently. There is also possible selection bias in our results due to incomplete cases and loss to follow‐up. Complete cohort cases differed from those lost to follow‐up across several important variables. Those with incomplete data were younger and more likely to have paid fees for services; the former could have resulted in underestimates of testing uptake. Since they did not differ by clinic, however, this bias should not have heavily influenced effect estimates reported here.

Second, the calculation of the cumulative facility index score has limitations. We were unable to calculate scores for visits to non‐Integra study facilities, and there were inconsistencies in recording of intervening facility visits between cohort rounds. At r1, information was captured on up to five intervening consultations, whereas information at r2 and r3 was restricted to the last visit. The effect of clinic exposure over time may therefore be underestimated in sites that would be more likely to encourage clients to come back more often.

Last, while efforts were made to remove duplicate reporting of HIV testing, our data cleaning indicated that respondents struggled to recall or report accurate HIV testing dates. Despite efforts to remove duplicate reports (e.g., where reported dates were very similar), there was still the possibility that tests reported in later rounds were duplications of tests reported earlier. Additionally, reporting bias may have increased over time with repeated survey rounds, thus potentially contributing to the markedly higher rates of testing over the course of the cohort. Reporting bias should not have differed between exposures, however.

## CONCLUSION

Assessing the impact of organizational changes on service outcomes is complex and sensitive to measurement definition choices. Using a range of measurements, our findings show that integrated delivery affects HIV testing goals if repeated contact with the integrated‐care delivery is sustained over time. Strategies to integrate HIV testing into FP services must therefore address sustained integrated delivery and encouragement of repeat service use by clients to ensure that they achieve their routine testing goals.

## Footnotes

1 The Integra Initiative is a registered clinical trial (No. NCT01694862) at clinicaltrials.gov.
